# A Comparative Study of Choroidal Vascular and Structural Characteristics of Typical Polypoidal Choroidal Vasculopathy and Polypoidal Choroidal Neovascularization: OCTA-Based Evaluation of Intervortex Venous Anastomosis

**DOI:** 10.3390/diagnostics13010138

**Published:** 2022-12-31

**Authors:** Figen Batıoğlu, Özge Yanık, Ferhad Özer, Sibel Demirel, Emin Özmert

**Affiliations:** Department of Ophthalmology, Ankara University School of Medicine, 06620 Ankara, Turkey

**Keywords:** choroidal vascularity index, indocyanine green angiography, optical coherence tomography angiography, polypoidal choroidal neovascularization, typical polypoidal choroidal vasculopathy, vortex vein anastomoses

## Abstract

Background: The aim of this study was to compare the choroidal characteristics of typical polypoidal choroidal vasculopathy (T-PCV) and polypoidal choroidal neovascularization (P-CNV) cases, and to investigate the presence of intervortex venous anastomoses in these PCV subtypes by using en face optical coherence tomography angiography (OCTA). Methods: A total of 35 eyes of 33 PCV cases were included. The PCV cases were divided into T-PCV and P-CNV groups. The choroidal vascularity index (CVI) was calculated. En face OCTA images were evaluated for the presence of intervortex venous anastomoses. The diameter of the largest anastomotic Haller vessel was measured. Results: T-PCV cases had significantly higher mean CVI values (73.9 ± 3.7 vs. 70.8 ± 4.5%) than P-CNV cases (*p* = 0.039). Intervortex venous anastomoses were observed in 85.7% of T-PCV eyes and in 91.7% of P-CNV eyes on en face OCTA (*p* = 1.000). In the cases with intervortex venous anastomosis, the mean diameter of the largest anastomotic vessel on en face OCTA was 341.2 ± 109.1 µm in the T-PCV and 280.4 ± 68.4 µm in the P-CNV group (*p* = 0.048). Conclusions: The higher CVI value in T-PCV may be an important feature concerning the pathogenesis and classification of PCV. Although there was no difference between the two subtypes in terms of intervortex anastomosis, more dilated anastomotic vessels were observed in the T-PCV.

## 1. Introduction

Polypoidal choroidal vasculopathy (PCV) is a disorder of the choroidal vasculature. Yannuzzi et al. first described this disorder in 1990, defining it as a distinct entity involving peculiar polypoidal, subretinal, vascular lesions associated with serous and hemorrhagic detachments of the retinal pigment epithelium (RPE) [1]. The main diagnostic features of the lesions are a branched network of choroidal vessels and polypoidal nodular structures on indocyanine green angiography (ICGA). Although PCV was initially considered a subtype of age-related macular degeneration (AMD), recent studies suggest that it belongs to the pachychoroid spectrum characterized by increased choroidal thickness, dilated Haller layer vessels, and choroidal hyperpermeability [2]. Current studies have described a novel finding, intervortex venous anastomosis, in pachychoroid-related disorders [3,4,5].

The advent of new imaging technologies has allowed for novel insight into the pathogenesis of PCV, revealing that polypoidal lesions are divided into two subtypes [6,7,8,9]. Yuzawa et al. reported that PCV cases could be classified as “typical PCV (T-PCV)” and “polypoidal choroidal neovascularization (P-CNV)” [9]. T-PCV cases have inner choroidal vessel abnormalities, whereas polypoidal CNV cases have CNV with polypoidal structures occurring at vessel termination points. Lower choroidal thickness, smaller polypoidal structures, and larger lesion area are the other discriminative features in P-CNV cases [10,11]. Later, Kawamura et al. suggested the terms type 1 PCV and type 2 PCV for P-CNV and T-PCV, respectively [12]. They also emphasized the importance of choroidal hyperpermeability in type 2 PCV. Tanaka et al. demonstrated a thicker choroid in type 2 PCV cases [13]. Coscas et al. divided PCV cases into neovascular AMD-related polyps and idiopathic polyps in 2015: Eyes with neovascular AMD-related polyps differ from idiopathic polyps by having larger lesions, thinner choroids, and more drusen [11]. Idiopathic polyps have the features of pachychoroids [11]. Therefore, grouping pachychoroid-driven PCV and AMD-related PCV under the same title may prevent the correct representation of these subtypes, as the underlying etiopathogenesis and choroidal structural characteristics may be quite different.

The development of sophisticated computer programs to binarize enhanced depth imaging mode optical coherence tomography (EDI-OCT) images has enabled the evaluation of the vascular structure of the choroid quantitatively and qualitatively. With the ability to evaluate the choroid as separate compartments, a novel term, the choroidal vascularity index (CVI), has been defined. The CVI represents proportionate changes in the choroidal vasculature. It has diagnostic importance and serves as a monitoring tool in choroidal disorders [14,15]. Although there are some studies reporting the CVI measurement in PCV cases, the results are controversial [16,17,18,19,20].

Additionally, recent development of ultra-wide-field ICGA has enabled better visualization of vortex veins, and current studies have proposed intervortex congestion and eventual anastomosis as keystones in pachychoroid spectrum disorder (PSD) pathophysiology [3,5,21,22]. Although it has been shown that anastomoses also exist in PCV, their role in the pathogenesis is still unknown [3].

Classifying PCVs with the abovementioned advanced imaging technologies would be helpful not only for pathogenic implications but also for prognostic significance [9]. Because the basic histopathological features of these two subtypes differ, the treatment response to therapeutic alternatives may also vary. In P-CNV, the main compartment of the lesion is the CNV; consequently, its natural course depends on the prognosis of the neovascular lesion. P-CNV has therefore been reported to show better response to antivascular endothelial growth factor injections in contrast to T-PCV [23]. However, photodynamic therapy has been reported to be a more effective treatment option in PCV cases with pachychoroid-driven conditions [10].

The aim of this study is to compare the choroidal structural characteristics of T-PCV and P-CNV cases, and to evaluate the presence of intervortex venous anastomoses in these subtypes. To the best of our knowledge, this is the first study comparing the presence of intervortex anastomoses and choroidal morphological features between these two distinct PCV subtypes.

## 2. Materials and Methods

This retrospective cross-sectional study included 35 eyes of 33 treatment-naïve PCV cases whose diagnosis was confirmed with indocyanine green angiography (ICGA) at the Retina-Vitreous Clinic of Ophthalmology at the Ankara University School of Medicine. This study was approved by the Institutional Review Board Committee of Ankara University and was conducted in accordance with the Declaration of Helsinki.

### 2.1. Patient Selection

The inclusion criteria were the detection of polypoidal lesions on ICGA images and presence of these lesions at subfoveal or juxtapapillary location, where the lesion remains on the 30-degree horizontal line scan through the fovea center. Eyes with any ocular inflammatory disease, other degenerative or vascular retinal conditions, or a spherical equivalent refractive error of ≥5 diopters were excluded. Those with a prior history of photocoagulation and/or vitreoretinal surgery were also excluded.

Demographic and clinical data of patients were obtained from their medical charts. A complete ophthalmic examination, including best corrected visual acuity (BCVA) measurement, slit-lamp biomicroscopy, and dilated posterior segment examination, were performed for all participants. Early Treatment of Diabetic Retinopathy Study (ETDRS) charts were used for BCVA assessment. The SPECTRALIS^®^ platform (Heidelberg Engineering, Heidelberg, Germany) was used for ICGA imaging. Spectral domain optical coherence tomography (OCT) images with the enhanced depth imaging (EDI) mode (SPECTRALIS^®^, Heidelberg Engineering) were used for choroidal measurements.

The definitive diagnosis of PCV was given based on ICGA images. Posterior pole 30° images and/or ultra-wide-field objective 102° imaging module ICGA images were used [5]. The PCV cases were classified into a T-PCV group (n = 23) and a P-CNV group (n = 12) according to the following criteria [7]. T-PCV was characterized by the presence of polypoidal lesions with/without branching vascular networks (BVNs). Polypoidal lesions were confirmed with ICGA showing early hyperfluorescent spots and BVNs. P-CNV was defined as the presence of smaller polypoidal structures affecting a limited area of a large type 1 macular neovascularization.

The presence of intervortex anastomosis at the watershed zone and asymmetry of the choroidal vessels between superior and inferior macula were evaluated using 6 × 6 mm en face OCTA (AngioVue software, RTVue XR Avanti, Optovue, Inc., Fremont, CA, USA). OCTA images could not be evaluated in 2 eyes due to motion artifacts. To standardize choroidal en face OCTA imaging, OCTA slabs, in which the largest Haller vessel could be detected below the parallel plane of the retinal pigment epithelial surface, were selected. Anastomoses between the superior and inferior vortex vein systems passing through the watershed zone in the macular area was defined as intervortex venous anastomosis. The PCV subtyping and evaluation of the presence of intervortex venous anastomosis were conducted by two retina specialists who were masked to the clinical data of the cases. In the case of conflicts regarding subtyping, a consensus on diagnosis was obtained after discussion. The diameter of the largest anastomotic vessel on en face OCTA was measured perpendicular to hyporeflective vessel walls in the region of the widest diameter of the lumen.

Subfoveal choroidal thickness, the maximum thickness from the outer portion of the hyperreflective line of the RPE and the inner surface of the chorioscleral junction, was measured on EDI-OCT scans by using the caliper tool of the SPECTRALIS^®^ software.

### 2.2. Choroidal Binarization

**Figure 1:**
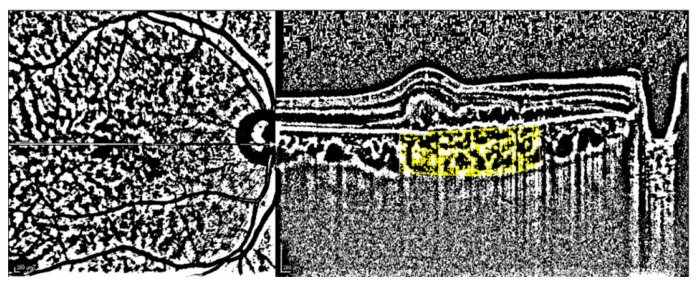
Binarized enhanced depth imaging mode OCT image using ImageJ software and Niblack autolocal thresholding.

ImageJ program version 1.52 u bundled with 64-bit Java 1.80_112 (Wayne Rasband, National Institutes of Health, Bethesda, MD, USA, https://imagej.nih.gov/ij accessed on 4 April 2020) was used for binarized choroidal measurements on EDI-OCT images of a horizontal B-scan through the fovea. Horizontal margins of 1500 μm from both sides of the fovea and vertical margins from the RPE to the choroidoscleral border were applied to measure the binarize the total choroidal area (CA). Niblack autolocal thresholding method was applied for binarization. The white pixels represented the stromal area (SA), and the black pixels represented the luminal area (LA). The choroidal vascularity index (CVI), the percentage of the LA to the total CA, was calculated (Figure 1).

The primary outcome measures were the total CA and the CVI values. The secondary outcome measure was the presence or absence of intervortex venous anastomosis on OCTA.

### 2.3. Statistical Analysis

Statistical Package for Social Sciences (SPSS) version 15.0 was used for the statistical analyzes. A Shapiro–Wilk test was performed for all variables as a test of normality. Continuous variables were expressed as the mean ± standard deviation (SD). Categorical variables were given as percentages (%). Depending on the normality of the data, a Mann–Whitney U test or an independent-samples T-test was used to compare the continuous variables between two PCV groups. A chi-square or Fisher’s exact test was used to evaluate the associations between categorical variables. Cohen’s kappa coefficient was calculated to quantify the intergrader agreement for PCV subtyping and intervortex venous anastomosis. A value of *p* <  0.05 was considered statistically significant.

## 3. Results

The T-PCV group included 23 eyes of 21 patients (11 males, 10 females), and the P-CNV group included 12 eyes of 12 patients (8 males, 4 females). The Cohen’s kappa coefficient between graders in determining the PCV subtype was almost perfect (0.806) [24]. A comparison of the demographic and ocular characteristics between the T-PCV and P-CNV groups is given in Table 1. The mean age was 67.5 ± 8.9 years in the T-PCV group and 66.1 ± 7.8 years in the P-CNV group (*p* = 0.655). The mean baseline BCVA score was 0.25 ± 0.30 LogMAR in the T-PCV group and 0.42 ± 0.29 LogMAR in the P-CNV group (*p* = 0.049).

**Figure 2:**
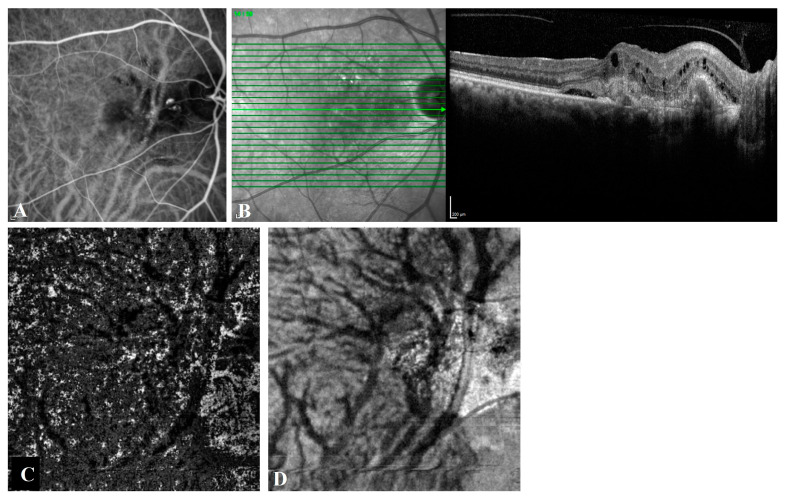
Angiographic and tomographic features of a 74-year-old female with T-PCV. (**A**) Posterior pole indocyanine green angiography shows dilated choroidal vessels passing through the horizontal watershed zone and hypercyanescent polypoidal lesions. (**B**) Spectral domain optical coherence tomography (OCT) reveals intraretinal cysts, subretinal hyperreflective material, subretinal fluid, shallow irregular PED, and a peaked PED corresponding to the polyp on ICGA. (**C**) En face OCT angiography and (**D**) en face OCT shows asymmetric congestion of inferotemporal vortex veins passing through the horizontal watershed zone at the level of the outer choroid, known as Haller’s layer.

A fundus examination revealed large serous pigment epithelial detachment (PED) in five (21.7%) of the T-PCV eyes and in one (8.3%) of the P-CNV eyes. The polypoidal lesions and the BVNs in the T-PCV group were in a juxtapapillary location (Figure 2) in thirteen eyes and in a subfoveal location in ten eyes. The CNV and its polypoidal dilatations in the P-CNV group were in a juxtapapillary location in 2 eyes and in a subfoveal location in 10 eyes. In the T-PCV group, 14 (60.9%) of the eyes had ≥300 µm subfoveal choroidal thickness, whereas only 3 (25.0%) of the eyes had ≥300 µm subfoveal choroidal thickness in the P-CNV group (*p* = 0.047). Among the eyes that had subfoveal choroidal thickness less than 300 µm, localized pachyvessels were observed in five of the eyes in the T-PCV group and in five of the eyes in the P-CNV group. Regarding quantitative choroidal measurements, the T-PCV group had significantly higher mean subfoveal choroidal thickness (356.0 ± 168.0 vs. 267.8 ± 68.7 µm) and CVI values (73.9 ± 3.7 vs. 70.8 ± 4.5%) compared to the P-CNV group (*p* = 0.036and *p* = 0.039, respectively).

**Figure 3:**
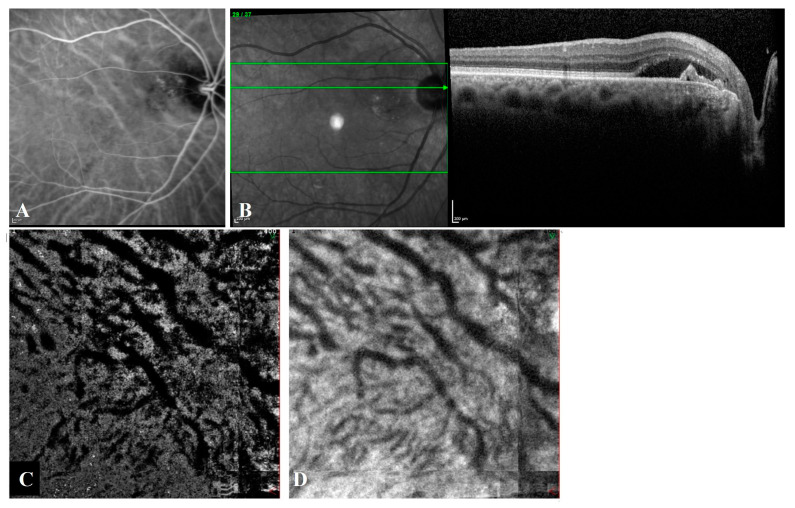
Angiographic and tomographic features of a 72-year-old female with T-PCV located at juxtapapillary area. (**A**) Indocyanine green angiography shows hypercyanescent polyps with a faint vascular network and large anastomotic choroidal veins coursing across the submacular region. (**B**) Spectral domain optical coherence tomography (OCT) reveals subretinal fluid with shallow irregular PED and a peaked PED corresponding to the branching vascular network and the polyp on ICGA. (**C**) En face optical coherence tomography angiography and (**D**) en face OCT shows asymmetric congestion of superotemporal vortex veins passing through the horizontal watershed zone at the level of the outer choroid, known as Haller’s layer.

**Figure 4:**
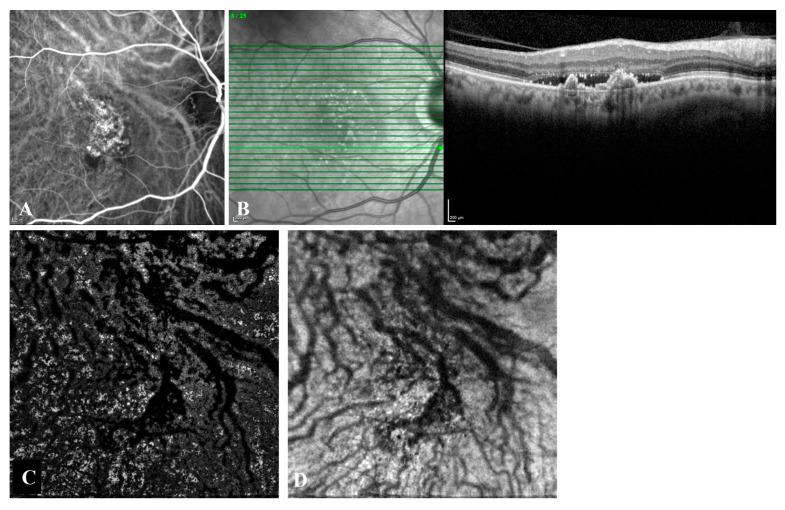
Angiographic and tomographic features of a 66-year-old male with subfoveal P-CNV. (**A**) Indocyanine green angiography shows hypercyanescent Type 1 CNV with polypoidal structures located at vessel termini. Large anastomotic choroidal veins coursing across the submacular area are clearly seen. (**B**) Spectral domain optical coherence tomography (OCT) reveals subretinal fluid and complex irregular PEDs. (**C**) En face optical coherence tomography angiography and (**D**) en face OCT shows asymmetric congestion of superotemporal vortex veins passing through the horizontal watershed zone at the level of the outer choroid, known as Haller’s layer.

Intervortex venous anastomoses were observed in 85.7% (18/21) of T-PCV eyes (Figure 3) and in 91.7% (11/12) of P-CNV eyes (Figure 4) on en face OCTA (*p* = 1.000). The Cohen’s kappa coefficient between graders in the detection of intervortex venous anastomosis was almost perfect (0.872) [24]. The mean diameter of the largest anastomotic vessel on en- face OCTA was 341.2 ± 109.1 µm in T-PCV and 280.4 ± 68.4 µm in P-CNV group (*p* = 0.048).

## 4. Discussion

The current nomenclature of PCV and its subclassifications remains controversial. PCV cases may present with a wide range of clinical features. Therefore, it is not clear if PCV is a distinct disease or may occur in different pathologic conditions leading to sub-RPE angiogenesis [25]. Various subclassifications based on imaging characteristics have been reported [8,11,26,27]. These studies propose different distinguishing features, including clinical features such as the configuration, location, or size of the polypoidal lesions or BVNs [28,29]. Subsequent studies also suggested the use of dye angiographies to detect leakage on fluorescein angiography or choroidal hyperpermeability on ICGA for subtyping [27,30]. However, no uniform classification has been formed yet. Therefore, it is still unclear whether PCV is a subset of AMD, an independent clinical entity, or a late-term consequence of longstanding pachychoroid disease.

Regardless of the subtype of PCV, detailed evaluation of the choroid is very important in PCV cases, as an abnormality in the choroidal vasculature is the underlying mechanism in the etiopathogenesis in all types. The CVI represents the quantitative evaluation of choroidal vascular and stromal areas. Basically, an increased subfoveal choroidal thickness contributes to a larger CA, and pathologically dilated choroidal vessels result in a larger LA and higher CVI values. There are a limited number of studies in which CVI measurement in PCV is performed, and the results are controversial [16,17,18,19,20]. A possible explanation of these controversial results is that the main title of “PCV” includes PCV subtypes in which CVI values may increase, such as in T-PCV, or decrease, such as in P-CNV [20]. Therefore, mean CVI values may appear as if they do not differ from normal controls, although they have different values in subtypes. For this reason, the accurate definition of PCV subtypes before undertaking a study is very important for revealing their choroidal characteristics in the best way. In our study, PCV subtypes were clearly determined at the beginning of the study, and analyses were performed according to these subtypes. We observed that T-PCV eyes had higher CVI values than P-CNV eyes, demonstrating the different degree of choroidal vascular involvement in the two subtypes. Similarly, Gupta et al. recruited choroidal features of patients with AMD and PCV from the prospectively planned Asian AMD Phenotyping Study, and indicated that two subtypes of PCV could be classified: T-PCV with increased choroidal vascularity and P-CNV with low choroidal vascularity based on the choroidal vascular features [20]. Another study, in which PCV subclassification was mainly based on choroidal hyperpermeability, found that the CVI in PCV without choroidal hyperpermeability was lower than the CVI in normal controls and in PCV cases with choroidal hyperpermeability [16].

The other studies evaluating the CVI in PCV did not include further subclassifications of PCV, and many focused on comparing the choroidal characteristics of PCV and AMD [17,18,19]. PCV eyes were reported to have lower CVI but a greater ratio of Haller’s layer to central choroidal thickness compared with the controls [19]. A 12-month prospective report indicated that PCV eyes with a high baseline CVI had significantly less stroma but a slightly larger LA than eyes with a low baseline CVI [17]. The authors claimed that the enlarged choroidal vessels in eyes with a high CVI could be accompanied by compressed or atrophied choroidal stroma. Moreover, Bakthavatsalam et al., studying PCV and CNV secondary to AMD, reported higher mean subfoveal choroidal thickness and greater LA values in PCV, but CVI values did not differ [18]. Gupta et al. found that the eyes with PCV without thick choroids had a similar choroidal vascular area to eyes with typical AMD [20].

Higher detection rates of veno-venous intervortex venous anastomosis in a great variety of PSDs, such as uncomplicated pachychoroid, central serous chorioretinopathy, peripapillary pachychoroid syndrome, and pachychoroid-associated neovascularization, have been reported [3,21]. Even the mean diameter of vortex veins was defined as a quantitative indicator of congestion [31]. Previously, Chung et al. demonstrated that engorgement of the vortex vein was observed more frequently in eyes with PCV [22]. Evaluation of ultra-wide-field ICGA images of patients with PCV showed higher vortex vein engorgement and larger areas of choroidal hyperpermeability compared to normal controls [32]. However, only one study has evaluated the presence of intervortex venous anastomosis in PCV. In that study, Matsumoto et al. [5] compared the frequency of anastomosis between the superior and inferior vortex veins in different PSDs and reported a higher detection rate in PCV (100%) compared to CSC (90.2%) and PNV (95.1%). They explained this increased frequency as a result of remodeling in the vortex veins due to longstanding asymmetric congestion of the choroid during the progression of pachychoroid spectrum disease [5]. In our study, we found the overall frequency of intervortex venous anastomosis as 87.9%. There is no statistically significant difference between the two subtypes in terms of intervortex anastomosis; however, more dilated anastomotic vessels were observed in the T-PCV. This difference was probably caused by the different degree of choroidal vascular involvement in the two subtypes. The enlarged choroidal vessels may explain both the higher CVI measurements and thicker anastomotic vessel diameter in T-PCV.

The major limitations of this study are its retrospective nature, the single-center and cross-sectional design, and relatively small number of PCV cases. Therefore, it is not known whether these anastomoses develop later or whether they occur at the beginning and then play a role in the process that will lead to the development of the disease. Further longitudinal studies with a larger sample size are needed to elucidate the precise pathogenesis of PCV.

## 5. Conclusions

In conclusion, the pathophysiology of PCV is still debated, especially regarding whether it is a distinct disorder from CNV or a disease with similar findings and polypoidal structures but with different vascular morphology. In our study, T-PCV and P-CNV differ, particularly in terms of choroidal vascular characteristics, which help to plan a more effective treatment approach by taking these morphological differences into consideration.

## Figures and Tables

**Table 1:** Comparisons of the demographic and ocular characteristics between typical polypoidal choroidal vasculopathy (T-PCV) and polypoidal choroidal neovascularization (P-CNV) groups.

	T-PCV (n = 23 Eyes)	P-CNV (n = 12 Eyes)	*p* Value
	Mean ± SD	Mean ± SD	
Age, years	67.5 ± 8.9	66.1 ± 7.8	0.655 ^a^
BCVA, LogMAR	0.25 ± 0.30	0.42 ± 0.29	*0.049* ^a^
Choroidal measurements			
Subfoveal choroidal thickness, µm	356.0 ± 168.0	267.8 ± 68.7	*0.036* ^b^
Total choroidal area, mm^2^	0.872 ± 0.392	0.708 ± 0.163	0.092 ^b^
Luminal area, mm^2^	0.647 ± 0.301	0.502 ± 0.122	0.052 ^b^
Stromal area, mm^2^	0.225 ± 0.096	0.206 ± 0.055	0.532 ^b^
Choroidal vascularity index, %	73.9 ± 3.7	70.8 ± 4.5	*0.039* ^b^
Indocyanine green angiography characteristics			
Total lesion area, mm^2^	4.917 ± 3.226	6.131 ± 5.946	0.797 ^a^
Optical coherence tomography angiography characteristics, n = 33			
Presence of intervortex venous anastomoses, %	85.7 (18/21)	91.7 (11/12)	1.000 ^c^

Statistically significant *p* values (<0.05) are italicized. ^a,b,c^ The tests used were Mann–Whitney U test, independent-samples T-test, and chi-square test, respectively. BCVA: best corrected visual acuity, SD: standard deviation.

## Data Availability

The datasets generated during and/or analyzed during the current study are available from the corresponding author on reasonable request.
